# Neutrophil-to-lymphocyte ratio for predicting palliative chemotherapy outcomes in advanced pancreatic cancer patients

**DOI:** 10.1002/cam4.204

**Published:** 2014-02-12

**Authors:** Peng Xue, Masashi Kanai, Yukiko Mori, Takafumi Nishimura, Norimitsu Uza, Yuzo Kodama, Yoshiya Kawaguchi, Kyoichi Takaori, Shigemi Matsumoto, Shinji Uemoto, Tsutomu Chiba

**Affiliations:** 1 Department of Clinical Oncology and Pharmacogenomics, Kyoto University Graduate School of MedicineKyoto, Japan; 2 Department of Medical Oncology and Shanghai Key Laboratory for Pancreatic Diseases, Shanghai First People's Hospital, Shanghai Jiaotong UniversityShanghai, China; 3 Department of Translational Clinical Oncology, Kyoto University Graduate School of MedicineKyoto, Japan; 4 Department of Gastroenterology and Hepatology, Kyoto University Graduate School of MedicineKyoto, Japan; 5 Department of Surgery, Kyoto University Graduate School of MedicineKyoto, Japan

**Keywords:** Chemotherapy, inflammation, NLR, pancreatic cancer, prognostic factor

## Abstract

Several previous studies reported that the neutrophil-to-lymphocyte ratio (NLR) could be a promising prognostic factor for patients with cancer. We aimed to determine the prognostic value of NLR in patients with advanced pancreatic cancer (APC) following palliative chemotherapy. We retrospectively reviewed 252 consecutive APC patients receiving palliative chemotherapy between January 2006 and December 2012. We classified the patients according to the pretreatment NLR values (≤5 or >5) into two groups and investigated the difference in treatment outcomes, including time to treatment failure (TTF) and overall survival (OS). A total of 212 patients had pretreatment NLR values of ≤5 (group A), while 40 patients had an NLR of >5 (group B). TTF and OS were significantly shorter in group B than in group A (3.1 vs. 8.7 months and 6.0 vs. 12.8 months, respectively; both *P* < 0.01). After adjustment for putative prognostic factors, including distant metastasis, status of recurrent/unresectable disease, pretreatment carbohydrate antigen 19-9 levels, and carcinoembryonic antigen levels using the Cox regression model, elevated pretreatment NLR remained an independent poor prognostic factor for OS (hazard ratio, 1.92; 95% confidence interval, 1.27–2.90; *P* < 0.01). In addition, patients in group B whose NLR dropped to ≤5 before the second cycle of chemotherapy showed longer TTF and OS compared with those whose NLR remained at >5. Our results support the idea that NLR can be a promising prognostic and predictive marker for APC patients receiving palliative chemotherapy.

## Introduction

Pancreatic cancer is one of the most lethal malignancies worldwide 1, and most patients are diagnosed too late for curative resection. Even with curative resection, disease relapse within 2 years occurs in >80% patients 2,3. Systemic gemcitabine-based chemotherapy has long been used as a standard therapy for patients with advanced pancreatic cancer (APC). However, the long-term efficiency and prognosis vary greatly among patients 4. Therefore, it is clinically relevant to identify APC patients who are more likely to benefit from palliative chemotherapy.

Accumulating evidence supports a positive relationship between inflammation and cancer development and progression 5,6. The interaction between tumor and host immune system promote tumor cell proliferation, metastasis, and also activate the inflammatory cascade in the host, which further deteriorates the general condition of cancer patients 6. Several markers, including neutrophil-to-lymphocyte ratio (NLR), platelet to lymphocyte ratio (PLR), and modified Glasgow prognostic score (mGPS), have been proposed to estimate the magnitude of systemic inflammation in cancer patients 7–9. Among these markers, a growing body of evidence supports the usefulness of NLR in predicting the prognosis of patients with cancer. Elevated NLR has reportedly been associated with poor survival following resection or chemotherapy in a variety of cancers 10–14. In pancreatic cancer, an increasing number of studies have reported an association between elevated NLR (>5) and poor prognosis 7,15–17. However, most studies included operable pancreatic cancer patients 7,15,18, and the prognostic value of NLR in APC patients receiving palliative chemotherapy is still limited. In fact, only one study of a relatively small cohort (*n* = 89) focused on APC patients receiving chemotherapy and demonstrated that elevated NLR could predict poor survival 16. Other studies that reported similar results analyzed the pooled data of patients who underwent surgery 17 or did not receive chemotherapy 7. Therefore, the usefulness of NLR as a prognostic marker for APC patients following chemotherapy should be validated in another large cohort. Furthermore, it is unknown whether the evaluation of NLR kinetics can predict outcomes for APC patients following chemotherapy.

In this study, we aimed to determine whether elevated NLR could be an independent poor prognostic factor in APC patients following chemotherapy and whether the monitoring of decreased NLR before the second cycle of chemotherapy could predict better outcomes.

## Patients and Methods

### Patients and treatment

Using a prospective cohort database system (CyberOncology®, Cyber Laboratory Inc., Tokyo, Japan) 19 and electronic medical charts, we retrieved the clinical data of 269 consecutive patients with pathologically confirmed pancreatic ductal adenocarcinoma who received at least two cycles of palliative first-line chemotherapy at Kyoto University Hospital (Kyoto, Japan) between January 2006 and December 2012. In principle, NLR was calculated using the neutrophils and lymphocytes counts obtained on the same day of chemotherapy. If blood test was not performed on the same day of chemotherapy, we substituted the data obtained within 2 days of chemotherapy. Sixteen cases were excluded from this study because a set of NLR values before the first and second chemotherapy cycles was not available, and 252 patients were ultimately investigated. Patients who had once undergone radical resection (R0 or R1) for primary tumors and developed recurrent disease were classified into the recurrent group (*n* = 73), while those who had an initial diagnosis of unresectable disease were placed into the initially unresectable group (*n* = 179). Palliative chemotherapy regimens included gemcitabine monotherapy (*n* = 156) 20, gemcitabine and S-1 combination therapy (*n* = 85) 21, S-1 monotherapy (*n* = 9) 22, and gemcitabine and erlotinib combination therapy (*n* = 2) 23. The standard doses and regimen schedules were adjusted at the discretion of the treating physicians according to incidence of adverse events or the general condition of the individual patient. All patients provided written informed consent for the use of their clinical data in the medical records system for research. This study was approved by the Ethics Committee of Kyoto University Graduate School of Medicine (E1606).

### Demographic/clinical and laboratory variables

Baseline patient characteristics, including laboratory data before the first cycle of palliative chemotherapy and the NLR values before the first and second cycles of chemotherapy, were collected for analysis. On the basis of previous studies,^24^–^26^ continuous parameters were categorized for the convenience of prognostic analysis as follows; age (<65 or ≥65 years), Eastern Cooperative Oncology Group Performance Status (ECOG PS) score (0–1 or 2), NLR (≤5 or >5), platelet to lymphocyte ratio (PLR) (<150 or ≥150), levels of carbohydrate antigen 19-9 (CA19-9, <1000 or ≥1000 U/mL), carcinoembryonic antigen (CEA, <5 or ≥5 ng/mL), C-reactive protein (CRP, <0.5 or ≥0.5 mg/dL), lactate dehydrogenase (LDH, <250 or ≥250 IU/L), hemoglobin (<10 or ≥10 g/dL), and albumin (<3.5 or ≥3.5 g/dL).

### Statistical analysis

Baseline patient characteristics were compared using the *χ*^2^ test or Fisher's exact test for dichotomous variables or the Mann–Whitney *U* test for continuous variables. The time to treatment failure (TTF) was calculated from the date of palliative chemotherapy initiation and terminated on the date of palliative chemotherapy discontinuation for various reasons, including treatment toxicity, disease progression, or patient withdrawal. Overall survival (OS) was calculated from the date of palliative chemotherapy initiation and terminated on the date of death for any reason or censored on the last follow-up visit. TTF and OS were estimated using the Kaplan–Meier method, and differences were compared using log-rank tests. Cox regression models were used to identify prognostic factors for TTF and OS. Prognostic factors shown to be significant in the univariate analysis were tested via multivariate analysis. The hazard ratio (HR) and 95% confidence interval (CI) were calculated using Cox regression models. A two-tailed *P* value of <0.05 was considered statistically significant. All statistical analyses were performed using SPSS statistical software (version 17.0; SPSS Inc., Chicago, IL).

## Results

### Patient characteristics

Patient characteristics were stratified by the pretreatment NLR values (≤5 or >5) and are summarized in Table 1. A total of 212 patients had a pretreatment NLR of ≤5, while 40 had an NLR of >5. Most baseline characteristics were comparable between the two groups. However, the following factors, including CA19-9 (≥1000 U/mL) levels, CRP (≥0.5 mg/dL) levels, LDH (≥250 IU/L) levels, and PLR (≥150) were more common in the NLR >5 group.

**Table 1:** Baseline characteristics.

Variables	Total (*n* = 252)	NLR ≤5 (*n* = 212)	NLR >5 (*n* = 40)	*P*-value
Age				
≥65	148 (58.7%)	122 (57.5%)	26 (65.0%)	0.48
<65	104 (41.3%)	90 (42.5%)	14 (35.0%)	
Gender				
Male	133 (52.8%)	110 (51.9%)	23 (57.5%)	0.61
Female	119 (47.2%)	102 (48.1%)	17 (42.5%)	
PS score				
0–1	242 (96.0%)	204 (96.2%)	38 (95.0%)	0.66
2	10 (4.0%)	8 (3.8%)	2 (5.0%)	
Distant metastasis				
Yes	184 (73.0%)	152 (71.7%)	32 (80.0%)	0.34
No	68 (27.0%)	60 (28.3%)	8 (20.0%)	
Primary tumor location				
Head	146 (57.9%)	127 (59.9%)	19 (47.5%)	0.16
Body and tail	106 (42.1%)	85 (40.1%)	21 (52.5%)	
The status of recurrent or unresectable				
Recurrent	73 (29.0%)	64 (30.2%)	9 (22.5%)	0.45
Unresectable	179 (71.0%)	148 (69.8%)	31 (77.5%)	
Palliative first line				
Gemcitabine monotherapy	156 (61.9%)	130 (61.3%)	26 (65.0%)	0.82
Gemcitabine and S-1	85 (33.7%)	73 (34.4%)	12 (30.0%)	
S-1 monotherapy	9 (3.6%)	7 (3.3%)	2 (5.0%)	
Gemcitabine and Erlotinib	2 (0.8%)	2 (1.0%)	0	
CA19-9 (U/mL)				
<1000	196 (77.8%)	170 (80.2%)	26 (65.0%)	0.04
≥1000	56 (22.2%)	42 (19.8%)	14 (35.0%)	
CEA (ng/mL)				
<5	145 (57.5%)	126 (59.4%)	19 (47.5%)	0.17
≥5	107 (42.5%)	86 (40.6%)	21 (52.5%)	
CRP (mg/dL)				
<0.5	175 (69.4%)	159 (75.0%)	16 (40.0%)	<0.01
≥0.5	77 (30.6%)	53 (25.0%)	24 (60.0%)	
LDH (IU/L)				
<250	219 (86.9%)	190 (89.6%)	29 (72.5%)	0.01
≥250	33 (13.1%)	22 (10.4%)	11 (27.5%)	
Hemoglobin (g/dL)				
<10	26 (10.3%)	20 (9.4%)	6 (15.0%)	0.27
≥10	226 (89.7%)	192 (90.6%)	34 (85.0%)	
Albumin (g/dL)				
≥3.5	183 (72.6%)	157 (74.1%)	26 (65.0%)	0.25
<3.5	69 (27.4%)	55 (25.9%)	14 (35.0%)	
PLR				
≥150	148 (58.7%)	110 (51.9%)	38 (95.0%)	<0.01
<150	104 (41.3%)	102 (48.1%)	2 (5.0%)	
TB (mg/dL)				
Median	0.7	0.7	0.7	0.87
Range	0.2–15.9	0.2–15.9	0.3–6.2	
AST (IU/L)				
Median	24	24	25	0.83
Range	11–466	11.00–466.00	11–122	
ALT (IU/L)				
Median	24	25	24	0.99
Range	7–564	8–564	7–250	
Creatinin (mg/dL)				
Median	0.7	0.7	0.7	0.34
Range	0.2–3.2	0.2–3.2	0.4–1.2	

### Prognostic factors for poorer TTF and OS

Univariate analysis identified eight prognostic factors associated with poorer TTF, including an ECOG PS of 2, distant metastasis, the status of unresectable disease, a pretreatment NLR of >5, CA19-9 levels of ≥1000 U/mL, CEA levels of ≥5 ng/mL, CRP levels of ≥0.5 mg/dL, and LDH levels of ≥250 IU/L. All these factors were subsequently analyzed in multivariate analysis. A total of five factors, including distant metastasis, status of unresectable disease, a pretreatment NLR of >5, CA19-9 levels of ≥1000 U/mL, and CRP levels of ≥0.5 mg/dL, remained independent prognostic factors for poorer TTF in APC patients following chemotherapy (Table 2).

**Table 2:** Univariate and multivariate analysis of poor prognostic factors for TTF.

		Univariate analysis			Multivariate analysis			
	*n*	Median TTF (95% CI) (months)	Hazard ratio	95% CI	*P*-value	Hazard ratio	95% CI	*P*-value
Age (years)								
≥65	148	7.7 (6.0–9.4)	1	0.74–1.27	0.83			
<65	104	8.0 (6.5–9.5)	0.97					
Gender								
Female	119	6.6 (5.0–8.2)	1	0.80–1.36	0.77			
Male	133	8.0 (6.4–9.6)	1.04					
ECOG PS								
0–1	242	7.4 (6.1–8.7)	1	1.03–3.68	0.04	1	0.84–3.15	0.15
2	10	2.2 (0.0–6.4)	1.95			1.62		
Distant metastasis								
No	68	9.0 (6.6–11.4)	1	1.28–2.44	<0.01	1	1.12–2.18	<0.01
Yes	184	6.9 (5.8–8.0)	1.77			1.56		
Primary tumor location								
Head	146	6.7 (5.7–7.7)	1	0.74–1.27	0.84			
Body and tail	106	9.3 (7.1–11.5)	0.97					
The status of initially unresectable/recurrent								
Recurrent	73	11.9 (7.2–16.6)	1	1.34–2.46	<0.01	1	1.17–2.20	<0.01
Initially unresectable	179	6.3 (4.9–7.7)	1.81			1.60		
NLR								
≤5	212	8.7 (7.2–10.2)	1	1.33–2.75	<0.01	1	1.08–2.31	0.02
>5	40	3.1 (2.7–3.5)	1.91			1.58		
PLR								
≤150	104	9.6 (6.8–12.4)	1	0.93–1.59	0.15			
≥150	148	6.3 (4.9–7.7)	1.22					
CA19-9 (U/mL)								
<1000	196	8.8 (7.2–10.4)	1	1.60–3.00	<0.01	1	1.10–2.21	0.01
≥1000	56	4.0 (2.2–5.8)	2.19			1.56		
CEA (ng/mL)								
<5	145	9.4 (7.3–11.5)	1	1.18–2.03	<0.01	1	0.99–1.76	0.06
≥5	107	6.2 (4.9–7.5)	1.55			1.32		
CRP (mg/dL)								
<0.5	175	8.8 (6.9–10.7)	1	1.40–2.47	<0.01	1	1.01–1.87	0.05
≥0.5	77	4.4 (2.8–6.0)	1.86			1.37		
LDH (IU/L)								
≥250	33	3.3 (2.0–4.6)	1	1.03–2.23	0.04	1	0.89–2.00	0.16
<250	219	7.9 (6.4–9.4)	1.51			1.34		
Hemoglobin (g/dL)								
≥10	226	7.5 (6.2–8.8)	1	0.74–1.75	0.57			
<10	26	5.1 (3.4–6.8)	1.13					
Albumin (g/dL)								
≥3.5	183	7.9 (6.3–9.5)	1	0.92–1.68	0.15			
<3.5	69	5.1 (2.4–7.8)	1.24					

The same analysis was performed for OS, and a total of five factors, including distant metastasis, status of unresectable disease, a pretreatment NLR of >5, CA19-9 levels of ≥1000 U/mL and CEA levels of ≥5 ng/mL, remained independent prognostic factors after multivariate analysis (Table 3).

**Table 3:** Univariate and multivariate analysis of poor prognostic factors for OS.

		Univariate analysis			Multivariate analysis			
	*n*	Median OS (95% CI) (months)	Hazard ratio	95% CI	*P*-value	Hazard ratio	95% CI	*P*-value
Age (years)								
≥65	148	12.1 (10.6–13.6)	1	0.74–1.29	0.87			
<65	104	11.3 (10.0–12.6)	0.98					
Gender								
Female	119	11.9 (10.5–13.3)	1	0.82–1.43	0.56			
Male	133	11.9 (10.0–13.8)	1.09					
ECOG PS								
0–1	242	12.0 (10.8–13.2)	1	1.09–3.92	0.02	1	0.91–3.46	0.09
2	10	4.4 (3.2–5.6)	2.07			1.78		
*Distant metastasis*								
No	68	16.7 (11.0–22.4)	1	1.49–2.98	<0.01	1	1.27–2.60	<0.01
Yes	184	11.2 (10.0–12.4)	2.11			1.81		
Primary tumor location								
Body and tail	106	12.2 (10.4–14.0)	1	0.72–1.26	0.72			
Head	146	11.2 (9.9–12.5)	0.95					
The status of initially unresectable/recurrent								
Recurrent	73	15.6 (10.9–20.3)	1	1.22–2.30	<0.01	1	1.08–2.12	0.02
Initially unresectable	179	11.1 (9.8–12.4)	1.67			1.51		
NLR								
≤5	212	12.8 (10.7–14.9)	1	1.50–3.15	<0.01	1	1.27–2.90	<0.01
>5	40	6.0 (2.8–9.2)	2.17			1.92		
PLR								
≤150	104	15.0 (13.3–16.7)	1	1.05–1.85	0.02	1	0.79–1.49	0.63
≥150	148	10.6 (9.6–11.6)	1.39			1.08		
CA19-9 (U/mL)								
<1000	196	13.4 (11.4–15.4)	1	1.78–3.45	<0.01	1	1.24–2.56	<0.01
≥1000	56	6.5 (4.6–8.4)	2.48			1.78		
CEA (ng/mL)								
<5	145	14.8 (12.5–17.1)	1	1.31–2.32	<0.01	1	1.11–2.04	0.01
≥5	107	10.1 (8.9–11.3)	1.74			1.50		
CRP (mg/dL)								
<0.5	175	13.4 (11.3–15.5)	1	1.37–2.48	<0.01	1	0.99–1.88	0.06
≥0.5	77	7.6 (4.6–10.6)	1.84			1.36		
LDH (IU/L)								
<250	219	12.3 (10.8–13.8)	1	1.00–2.22	0.05	1	0.84–1.98	0.24
≥250	33	6.6 (2.7–10.5)	1.49			1.29		
Hemoglobin (g/dL)								
≥10	226	12.0 (10.6–13.4)	1	0.74–1.88	0.48			
<10	26	9.6 (5.7–13.5)	1.18					
Albumin (g/dL)								
≥3.5	183	12.2 (10.6–13.8)	1	0.99–1.83	0.06			
<3.5	69	10.0 (6.7–13.3)	1.34					

### Relationship between NLR thresholds and OS

Table 4 shows the relationship between different thresholds of NLR and OS. An NLR cutoff value of 5 could discriminate patients with poorer survival and the highest HR in our cohort.

**Table 4:** The NLR thresholds and relationship with survival.

		Univariate analysis			Multivariate analysis^1^				
	*n* (%)	Median OS (95% CI) (months)	Hazard ratio	95% CI	*P*-value	Hazard ratio	95% CI	*P*-value	
NLR	≤1	14 (5.6)	12.8 (9.4–16.2)	1	0.86–3.29	0.13	1	0.69–2.71	0.37
	>1	238 (94.4)	11.7 (10.4–13.0)	1.68			1.37		
	≤2	83 (32.9)	14.8 (11.5–18.2)	1	1.13–2.05	0.01	1	0.88–1.66	0.24
	>2	169 (67.1)	10.7 (9.3–12.1)	1.52			1.21		
	≤3	158 (62.7)	13.4 (11.1–15.7)	1	1.26–2.23	<0.01	1	1.18–2.11	<0.01
	>3	94 (37.3)	8.6 (6.2–11.0)	1.68			1.57		
	≤4	194 (77.0)	13.3 (11.4–15.2)	1	1.44–2.78	<0.01	1	1.36–2.67	<0.01
	>4	58 (23.0)	7.3 (5.6–9.0)	2.00			1.91		
	≤5	212 (84.1)	12.8 (10.7–14.9)	1	1.50–3.15	<0.01	1	1.49–3.15	<0.01
	>5	40 (15.9)	6.0 (2.8–9.2)	2.17			2.16		

1 Multivariable analysis was adjusted for distant metastasis, status of recurrent, CA19-9, and CEA.

### Comparison of TTF and OS stratified by pretreatment NLR

**Figure 1:**
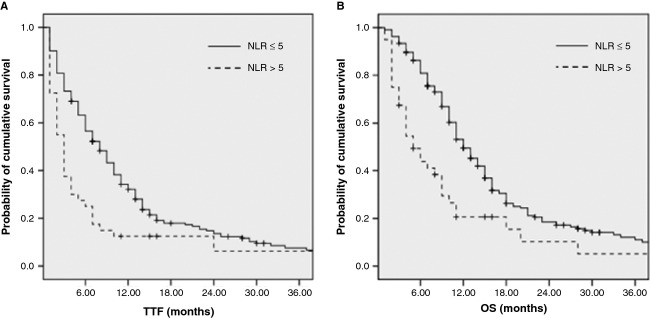
TTF (A) and OS (B) according to basal NLR in APC patients following palliative chemotherapy.

The median TTF and OS in patients with a pretreatment NLR of >5 was 3.1 (95% CI, 2.7−3.5) months and 6.0 (95% CI, 2.8–9.2) months, respectively, which were significantly shorter compared with those of patients with an NLR of ≤5 (TTF and OS, 8.7 [95% CI, 7.2−10.2] months and 12.8 [95% CI, 10.7−14.9] months, respectively; both *P* < 0.01; Fig. 1A and B).

### NLR drop (≤5) before the second cycle of chemotherapy predicted favorable TTF and OS

**Figure 2:**
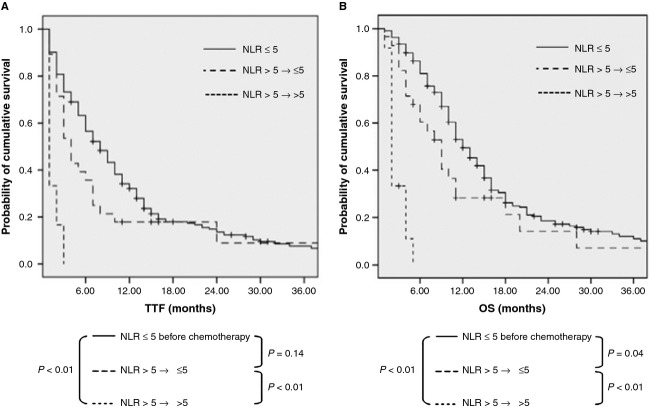
TTF (A) and OS (B) according to NLR change before the second cycle of chemotherapy in APC.

To test whether the monitoring of the drop in NLR before the second cycle of chemotherapy could predict better outcomes, patients with a pretreatment NLR of >5 were categorized into two groups according to their NLR levels before the first and second cycles of chemotherapy as follows: group 1, NLR >5 at baseline and drop to ≤5 before the second cycle of chemotherapy (*n* = 28); and group 2, NLR >5 before both the first and second cycles of chemotherapy (*n* = 12). Patients in group 1 demonstrated significantly improved TTF and OS compared with those in group 2 (4.3 vs. 1.4 months and 9.3 vs. 2.7 months, respectively; both *P* < 0.01; Fig. 2A and B).

## Discussion

Growing evidence supports a positive relationship between inflammation and cancer development and progression 5,6. NLR is attracting more and more researchers' attention because it is readily measurable in peripheral blood and is likely to reflect the magnitude of the systemic inflammatory response. An increasing number of studies have reported that elevated NLR can be a marker of poorer prognosis in a variety of cancers 10–14. Elevated NLR is often accompanied by elevated neutrophil levels and relative lymphocytopenia. Elevated neutrophil levels can promote tumor cell progression by upregulating a variety of inflammatory cytokines and providing a suitable microenvironment for tumor growth 27,28. Furthermore, lymphocytopenia arising from numerous inhibitory immunologic mediators released by tumor cells represents an immunosuppressive condition in cancer patients and contributes to poorer outcome 29.

In this study, we aimed to determine whether elevated pretreatment NLR was associated with poorer prognosis for APC patients receiving palliative chemotherapy. Cox regression analysis identified a total of five factors, including distant metastasis, status of unresectable disease, a pretreatment NLR of >5, CA19-9 levels of ≥1000 U/mL, and CEA levels of ≥5 ng/mL, that were associated with poorer OS in our cohort. We observed significantly shorter TTF and OS among patients with a pretreatment NLR of >5 compared with those among patients with an NLR of ≤5. The median OS was 6.0 months in patients with an NLR of >5 and 12.8 months in patients with an NLR of ≤5. In addition, the NLR cutoff value of 5 was determined to be optimal in our cohort. Dexamethasone is commonly used for antiemetic purpose in systemic chemotherapy; however, the mean dose of dexamethasone used for antiemetic purpose was almost equal (2.2 mg) between group A and group B and it was unlikely that this affected our current results. The present results are in line with those of previous studies 16,17 reporting that elevated NLR was an independent prognostic factor for OS in APC patients receiving palliative chemotherapy; these data from published studies are summarized in Table 5. The proportion of patients with a pretreatment NLR of >5 in existing research are comparable across studies. To the best of our knowledge, our current study comprised the largest number of APC patients who received palliative chemotherapy, and our results strongly support the hypothesis that elevated NLR (>5) can be a reliable and reproducible marker for identifying a subgroup of APC patients with poorer prognosis following palliative chemotherapy.

**Table 5:** Summary of published studies reporting the association between NLR and the prognosis of APC patients receiving chemotherapy.

Study	Year	*n*	Number of patients with NLR >5 (%)	Overall survival (NLR >5 vs. ≤5) (months)	Hazard ratio (NLR ≤5 was set at 1)
An X et al. 16	2010	89	16 (17.9)	2.4 versus 7.7	HR = 4.49, *P* = 0.013
Wang DS et al. 17	2012	86	12 (13.9)	5.8 versus 10.2	NA
Stotz M et al^1^ ^7^	2013	261	79 (30.3)	NA	HR = 2.53, *P* < 0.01
Our study	2013	253	40 (15.8)	6.0 versus 12.8	HR = 1.95, *P* < 0.01

NA, not available.

1 This study (*n* = 261) pooled the data from patients who received chemotherapy (*n* = 179) and no chemotherapy (*n* = 82).

We also demonstrated that NLR kinetics could predict treatment outcome in APC patients following palliative chemotherapy. Patients whose pretreatment NLR values of >5 dropped to ≤5 before the second cycle of chemotherapy demonstrated significantly longer TTF and OS compared with those whose NLR values remained at >5 before the second cycle of chemotherapy. A total of five patients developed grade 3 or higher neutropenia during the first cycle of chemotherapy in group B. A persistent NLR of >5 before the second cycle of chemotherapy remained an independent poor predictive marker of TTF and OS (both *P* < 0.01) after adjusting the incidence of grade 3 or higher neutropenia during the first cycle of chemotherapy. Persistent elevation of NLR may reflect the severe systemic inflammatory response in the body and aggressive tumor features. Our results are in line with those of the previous study by Chua et al. 11 They investigated a total of 162 patients with metastatic colorectal cancer who received palliative chemotherapy and reported that patients whose pretreatment NLR values of >5 dropped to ≤5 before the second chemotherapy cycle demonstrated significantly longer progression-free survival and a trend toward longer OS compared with patients with a persistent NLR of >5. Therefore, evaluation of NLR before the second cycle of chemotherapy can help physicians to predict chemotherapy resistance and reconsider the treatment strategy at an earlier time point in daily clinical practice.

In contrast to NLR, we were unable to validate the prognostic value of PLR or mGPS in our cohort, although some researchers reported that these play prognostic roles in patients with cancer 8,9. This study was limited by its retrospective design. In addition, chemotherapy regimens differed among patients; however, it is unlikely that chemotherapy regimen heterogeneity affected the current results because almost 99% patients received gemcitabine, S-1, or gemcitabine/S-1 combination therapy, and the efficacies of these three regimens were not statistically different in a large randomized phase III study 30.

In summary, our results strongly support the idea that NLR can be a promising prognostic marker for APC patients receiving palliative chemotherapy. Furthermore, evaluation of NLR before the second cycle of chemotherapy can help physicians to predict response to palliative chemotherapy at an earlier time point. Future prospective studies are warranted to verify the usefulness of monitoring NLR in treating patients with APC.
